# Editorial: Comparative Immunology of Marine Mammals

**DOI:** 10.3389/fimmu.2019.02300

**Published:** 2019-10-01

**Authors:** Giovanni Di Guardo, Michael Frederick Criscitiello, Eva Sierra, Sandro Mazzariol

**Affiliations:** ^1^Faculty of Veterinary Medicine, University of Teramo, Teramo, Italy; ^2^Department of Veterinary Pathobiology, College of Veterinary Medicine and Biomedical Sciences, Texas A&M University, College Station, TX, United States; ^3^Faculty of Veterinary Medicine, Instituto Universitario de Sanidad Animal y Seguridad Alimentaria, University of Las Palmas de Gran Canaria, Las Palmas de Gran Canaria, Spain; ^4^Department of Comparative Biomedicine and Food Science, University of Padua, Padua, Italy

**Keywords:** comparative immunology, innate immunity, acquired immunity, host–pathogen interaction, immunotoxic contaminants, marine mammals, cetaceans, pinnipeds

Marine mammals rank among the most charismatic creatures inhabiting our planet. Yet despite their long evolutionary history, we have a limited knowledge of their biology, ecology, and behavior, including their morphofunctional adaptations to sea life. This is also frustrating for translational studies, as gas and fat embolic syndrome, a pathologic condition of *Ziphiidae* (deep-diving cetaceans), mimics decompression sickness (DCS) in human divers (1). Indeed, the development of a DCS-like condition in animals living exclusively in a marine environment for such a long evolutionary history was totally unpredictable!

Marine mammals, comprising cetaceans, pinnipeds, manatees, sea otters, and polar bears, are all included in the International Union for the Conservation of Nature (IUCN) Red List, being increasingly threatened worldwide by several anthropogenic and natural factors, often acting synergistically with each other, as in the case of infectious agents and immunotoxic environmental pollutants (2), high levels of which may be found in tissues from stranded cetaceans, especially dolphins, given their recognized role of “apex predators” (3). Despite the increased efforts made by the scientific community, a considerable knowledge gap still exists on many aspects of marine mammals' biology. This is especially true regarding their immune response(s) at both individual and population levels, an absolute prerequisite to understand their immunopathological counterparts (i.e., immunodeficiencies, hypersensitivity reactions, and autoimmunity), impaired by a substantial amount of data coming from studies performed on stranded animals, which are “not normal” by definition. Within such context, primary fibroblast cell cultures from skin biopsies of free-living pinnipeds and cetaceans have marked a significant progress in our capability of monitoring the effects of a wide range of persistent environmental pollutants on marine mammals' health, including their stress and immune response genetic control (4).

Still noteworthy, aquatic mammals are increasingly threatened by several infectious *noxae* like morbilliviruses, herpesviruses, *Brucella ceti, B. pinnipedialis*, and *Toxoplasma gondii*, just to cite a few (2). Indeed, cetacean morbillivirus (CeMV), phocine distemper virus (PDV), and canine distemper virus (CDV) have recently caused several mass die-offs among dolphin, whale, and seal populations across the globe (5, 6). Consequently, characterizing the host- and the virus-related factors driving the occurrence, the behavior and persistence of morbilliviral infection(s) inside a given marine mammal species and/or population are of paramount importance (7), together with the study of host innate and acquired antiviral immunity. No information is available, for instance, on the pathogenetic evolution of CeMV, PDV, and CDV infections in Th1-dominant vs. Th2-dominant individuals within susceptible dolphin, whale, and seal species (8). In this respect, *ad hoc* investigations on human immunodeficiency virus have clearly shown that Th2-dominant patients are much more prone than Th1-dominant individuals to develop full-blown AIDS (9). Studying the host's antiviral immunity could also shed light on the “transmission barrier's jump,” which has been repeatedly observed across the Western Mediterranean, in recent years, from CeMV-infected “donor” species (like striped dolphins, *Stenella coeruleoalba*) to “recipient” species like fin whales (*Balaenoptera physalus*) (10), sperm whales (*Physeter macrocephalus*) (11), Cuvier's beaked whales (*Ziphius cavirostris*) (12), and even common seals (*Phoca vitulina*) (13) and Eurasian otters (*Lutra lutra*) (14). Within such context, the possibility that CeMV infection may also spread transplacentally is of concern, given the less efficient immune response during pregnancy (15). A defective functioning of innate and acquired immunity could also represent a key factor underlying viral persistence inside the host's brain, exemplified by the peculiar “brain-only” forms of morbilliviral disease reported in CeMV-infected striped dolphins, which share neuropathologic similarities with subacute sclerosing panencephalitis in measles virus-infected humans and with old dog encephalitis in CDV-infected canines (8). Deepening our knowledge on the comparative immunology of marine mammals may additionally provide valuable insights into the interplay between the infectious pathogens of concern to and the microbiota of pinnipeds, cetaceans, and sea carnivores, on one side, and a wide range of immunotoxic pollutants, which may heavily accumulate inside their tissues, on the other (2, 3). These high contaminant tissue loads could also exert a cell-transforming activity, as clearly shown in the beluga whale (*Delphinapterus leucas*) population residing in the St. Lawrence River Estuary, Canada, in which neoplastic disease was found in association with high tissue burdens of organochlorines, heavy metals, and benzo-a-pyrene. A direct and an indirect carcinogenic activity could have both been involved in the development of the aforementioned neoplasms, together with an antitumor immune surveillance deficiency (16).

A critical component of all studies on the comparative immunology of marine mammals is that the vast majority of the laboratory reagents available on the market have been developed and “validated” only in “conventional” species. Therefore, *ad hoc* immunologic tools, specifically developed and/or validated also for marine mammals, are urgently needed.

The aforementioned issues represent the focus, along with several others, of the present Research Topic on the comparative immunology of marine mammals, hosting eight original contributions from leading scientists and scientific groups worldwide. In more detail, the monograph's contents address T-helper lymphocytes and other cellular and molecular effectors of cetaceans' immune response, acute-phase proteins and class II major histocompatibility complex in marine mammals, along with antioncogenic virus immune surveillance in California sea lions (*Zalophus californianus*), cetacean host–pathogen interaction(s), and the comparative immunopathology of CeMV infection in free-ranging dolphins. In this respect, we would like to express our most sincere feelings of appreciation and gratitude to Prof. Miki Nakao, chief editor of *Frontiers in Immunology*, who enthusiastically accepted our proposal to host this monographic collection in such an outstanding and prestigious Journal, as well as to Dr. Tara Sugrue, Research Topic team leader, Dr. Carmen E. Flores Nakandakare, and all the eminent scientists, associate editors, and reviewers who provided their valuable contributions for the present Research Topic.

## Author Contributions

GD wrote the first draft of the present Guest Editorial, which was subsequently revised by MC as well as by ES and SM, who provided their valuable and precious comments, remarks, and suggestions. The final Editorial's draft and the current Editorial's text were entirely agreed and approved by all the 4 Guest co-Editors of the present Research Topic, dealing with the Comparative Immunology of Marine Mammals.

### Conflict of Interest

The authors declare that the research was conducted in the absence of any commercial or financial relationships that could be construed as a potential conflict of interest.
